# The contribution of water extractable forms of plant nutrients to evaluate MSW compost maturity: a case study

**DOI:** 10.1038/s41598-020-69860-9

**Published:** 2020-07-30

**Authors:** Elzbieta Jamroz, Jakub Bekier, Agnieszka Medynska-Juraszek, Andrea Kaluza-Haladyn, Irmina Cwielag-Piasecka, Magdalena Bednik

**Affiliations:** Institute of Soil Science and Environmental Protection, Wroclaw University of Environmental and Life Sciences, ul. Grunwaldzka 53, 50-357 Wrocław, Poland

**Keywords:** Environmental sciences, Natural hazards

## Abstract

The object of the experiment was to evaluate municipal solid waste (MSW) compost. Composting was carried out in a pile under aerobic conditions. Total content as well as water-extractable forms of macro and microelements were analysed during composting. Nutrient solubility indices were calculated for samples taken at various stages of maturity. The soluble forms of C, P, K, Ca and Mg decreased relatively to their total forms following maturation phases. For all micronutrients tested, a significant reduction in the proportion of soluble forms in relation to their total content was observed with an increase in composting time. In mature compost, low solubility were found for nitrogen, potassium, sodium and magnesium, which may indicate that the final product is a good source of these nutrients. The solubility index (percentage share of water-extractable forms of macro- and micronutrients in the total content) for iron indicates that the composting process does not affect its degree of solubility. Solubility index instead of the content of water-extractable forms of chosen macro- and microelements could be taken into account in determining the degree of MSW compost maturity.

## Introduction

Compost obtained from waste biomass as well as from municipal solid waste is a good source of stable organic matter and, due to the presence of good-quality nutrients, can be efficiently used in agriculture^1–3^. Organic waste contains a high amount of organic carbon (30–50%) and easily available nitrogen and thus should be utilized particularly in intensively cultivated areas^4–7^. During the composting process, organic matter undergoes stabilization and humification^8,9^ and the final product (matured compost) is widely described as a good amendment to improve the physical, physico-chemical, chemical and biological properties of soils and as a consequence increases plant yield^10–15^. On the other hand, municipal solid waste (MSW) compost may contain variable concentrations of potentially harmful components, particularly metals and metalloids, thus its application to soil is in many countries subject to limitations by law. Application to soil of MSW compost containing a high amount of metal(loids), for example As, may be the reason for increasing accumulation in plants and consequently cause adverse effects on the environment—soil and water contamination^3,16–19^. Most studies point to the composting process and degree of maturity of the final product as basic conditions for its safe utilization. Application to soil of unstable and immature compost inhibits seed germination, reduces plant growth and causes phytotoxicity to plants^20–23^.

The water-extractable components of composting material have been described as being more easily utilized by microbes and more bioavailable in comparison to solid-phase components during the composting process^14,24–28^. Moreover, some authors claim that characterization of the water-extractable phase is a better indicator of the overall transformation of organic matter during the composting process than characterization of the solid phase^24,28,29^. During the course of the composting process, changes are observed in the concentrations of macro- and microelements soluble in water. Leita and De Nobili^30^ analysed the process for 60 days and found that an increase in total heavy metal concentrations during composting was not accompanied by an increase in the concentrations of water-extractable metals. They also did not find any relation between water-soluble metal concentration and pH. On the other hand, various authors have observed a significant correlation between compost stability and dissolved organic matter^31,32^. More labile compounds, i.e. water-extractable ones, are a source of carbon and energy for the microbial biomass, thus their concentration decreases rapidly with an increase in composting time^26,30,33^.

There are still only a few papers dealing with the proportion of water-soluble macro- and microelements in relation to the total content during the composting process, so the authors decided to present the results of an experiment with compost produced from MSW and the concentration of the water-extractable fractions of some macro- and microelements.

The aim of the work was to improve knowledge of MSW composting through analysis of the water-soluble phase and attempt to establish new compost maturity indices based on the water-soluble forms of macro- and/or microelements.

## Material and methods

### Composting process and sampling

**Figure 1:**
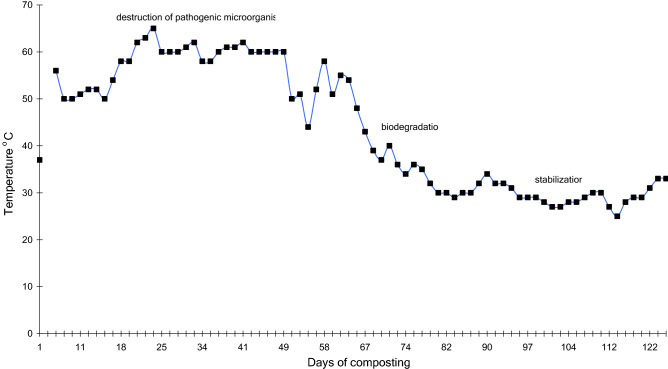
Changes of temperature during composting of municipal solid wastes.

The object of the experiment was municipal solid waste (mixed wastes) compost (MSWC) from the Katowice agglomeration, located in the Upper Silesian region of southern Poland (50.293238, 19.069045). Raw material from the composting plant working according to DANO technology^34^ was composted for 279 days. The main technological device in the DANO system is a biostabilizer, which consists of two basic elements determining the course of composting: a closed fermentation chamber, in which optimal conditions prevail for an intensive course of technological processes, and a slow-speed drum crusher, in which waste dispersion occurs through abrasion and tearing. After 36 h from the introduction of waste into the biostabilizer, raw material is obtained. Further composting takes place in piles in the open air. In the experiment composting was carried out in a pile under aerobic conditions. The composting pile was 4 m long, 2 m wide and 1.5 m high. Composted material was mixed every 10–15 days, the moisture was maintained at 50% w/w and the temperature was measured daily (Fig. 1). Samples were taken every 10–15 days from about 20 places, then mixed, and the final weight of the sample was 2.5–3 kg of fresh material. All contaminants (plastic, glass, metal, etc.) were removed. Samples were dried at room temperature, then ground and sieved (1 mm). Total organic carbon (TOC) was analysed with a CS-mat 5500 instrument (Strohlein GmbH & Co., Kaarst, Germany, currently Bruker AXS Inc., Madison, WI, USA), total nitrogen (TN) by the Kjeldahl method using a Buchi Labortechnik GmbH N analyser, and cation exchange capacity (CEC) by the Harada and Inoko method^35^.

### Aqueous compost extracts

Aqueous compost extracts were prepared by placing 100 g (dry weight) of compost at different maturity stages in 100 ml of distilled water and shaking for 2 h at room temperature. The suspension was filtered and some macro- (water-extractable macroelements—WEMAE) and microelements (water-extractable microelements—WEMIE) in the filtrate were analysed: pH was measured by the potentiometric method, electrical conductivity (EC) was measured using a conductivity meter (Mettler Toledo Seven Multi 2009) and water-extractable carbon (C_w_) was analysed with a CS-mat 5500 instrument (Strohlein GmbH & Co., Kaarst, Germany, currently Bruker AXS Inc., Madison, WI, USA). Water-extractable nitrogen (N_w_) was analysed by the Kjeldahl method using a Buchi Labortechnik nitrogen analyser; K_w_, Ca_w_, Mg_w_, Cu_w_, Zn_w_, Mn_w_, Fe_w_, Pb_w_, Cd_w_, Cr_w_ and Ni_w_ were determined using FAAS; the N-NH_4_ and N-NO_3_ content was analysed using a SAN^++^ SFA continuous flow colorimeter (Skalar Analytical B.V., Holland).

The solubility index (*I*), presented as the percentage share of water-extractable forms of macro- and micronutrients in the total content, was calculated according to Eq. (1):

1 $$Ip = \, (pw/pt) \, \times { 1}00$$

p—macro- or microelement, pw—water-extractable form of element, pt—total content of the element.

### Statistical analysis

Results were statistically verified using Statistica 12.5. Means were compared by the Tukey test, at a confidence level of P < 0.05.

## Results and discussion

### pH and EC during composting

Changes in H^+^ concentration were observed during composting of MSW, reflected by analysis of water solution pH (Table 1). The lowest pH values (6.9) were found for water extracts from raw material. During the composting process, the pH increased, with the highest values being observed at between 54 and 126 days. In the following phases of the composting process, a slight decrease in pH was observed (8.0–8.1), but the result was significantly higher than those observed in the first days of the composting process. The decomposition of organic matter, particularly its components like proteins, amino acids and peptides, causes the release of ammonium which contributes to an increase in pH^36^. Changes in the EC of water extracts during the composting process were observed as well (Table 1). The values of this parameter depended on the length of the composting process, significantly decreasing with an increased in composting time. After about 54–70 days of composting, the EC value was about 2 mS/cm, and in the final phase it increased slightly to 2.15 mS/cm. From the 54th to 68th day of composting, we observed some stabilization of EC, and lower values with respect to that of the initial material, indicating a reduction in the amount of soluble salts in the matured compost. The results obtained, according to many authors^36,37^, suggest that the concentrations of soluble salts in the compost analysed here were in the range considered non-phytotoxic.

**Table 1:** Basic parameters of the municipal solid waste compost used in the experiment during the process of maturation.

Parameter	Days of composting													
	1	11	22	36	54	68	82	95	112	126	143	159	188	279
TOC g kg^-1^	285.9^a^	258.0^b^	194.1^c^	177.3^d^	168.7^e^	165.2^e^	163.3^e,f^	158.3^g^	156.8^g^	155.5^g^	150.3^g^	146.7^h^	145.3^h^	144.3^h^
Nt g kg^-1^	9.8^a^	10.1^a^	10.8^b,a^	11.5^b^	12.1^b^	11.9^b^	12.7^b,c^	12.5^b,c^	12.9^b,c^	13.0^b,c^	13.2^c^	13.0^b,c^	12.9^b,c^	12.9^b,c^
C/N	29.3^a^	25.5^b^	18.0^c^	15.4^d^	13.9^e^	13.9^e^	12.8^e^	12.7^e^	12.1^e,f^	11.9^f,g^	11.4^e,f^	11.3^e,f^	11.3^e,f^	11.2^e,f^
CEC dmol( +) kg^-1^	37.7^a^	46.2^b^	47.0^b^	54.7^c^	62.2^d^	59.7^e^	65.1^f^	65.6^f^	67.0^f,g^	67.9^g^	73.6^h^	73.2^h^	76.5^i^	80.0^j^
pH	6.9^a^	6.9^a^	6.9^a^	7.5^b^	7.9^b^	7.7^b^	8.3^b,c^	7.8^b^	8.1^b,c^	8.3^b,c^	8.0^b^	7.7^b^	8.0^b^	8.1^b,c^
EC mS cm^-1^	3.70^a^	3.20^b^	3.25^b^	2.48^c^	2.00^d^	1.98^d^	2.25^d,e^	2.20^c,d^	2.10^c,d^	2.40^c,d^	2.15^c,d^	2.00^d^	1.60^d^	2.15^c,d^
Ash g kg^-1^	427.9	479.2	540.1	572.5	585.3	593.9	594.8	603.2	606.5	607.0	607.4	613.1	610.9	637.2

Means followed by the same letter are not significantly different at p < 0.05.

### Change of WEMAE

During the experiment, a significant correlation was observed between the amount of macroelements soluble in water and the composting time. The length of composting significantly reduced the amount of water-soluble forms of most macroelements. The C_w_ content significantly decreased from 14.1 g/kg in the raw material to 1.2 g/kg after the composting process ended (Table 2). The reduction of carbon solubility during the composting process is explained by many authors^24,36,38^ as a biochemical transformation of organic matter. Metabolites of microorganisms are manifested in the water-soluble phase. The easily soluble nutrients are absorbed by the microorganisms and the insoluble forms (of high molecular weight) are converted by microbial enzymes into soluble forms and consistently absorbed by their cells.

**Table 2:** Changes of water-extractable forms of carbon and nitrogen during composting of municipal solid wastes.

Parameter	Days of composting													
	1	11	22	36	54	68	82	95	112	126	143	159	188	279
C_w_ g kg^-1^ d.m	14.100^a^	10.440^b^	7.100^c^	9.790^d^	3.620^e^	3.020^f^	2.340^g^	1.650^h^	1.490^h^	1.390^h^	1.140^h^	1.270^h^	1.320^h^	1.200^h^
N_w_ g kg^-1^ d.m	1.762^a^	1.626^a^	1.333^a,b^	1.471^a^	0.882^c^	0.642^c^	0.725^c^	0.839^c^	0.942^c^	1.162^a,c,d^	1.043^b,c,d^	1.122^b,c,d^	1.093^b,c,d^	1.118^a,b,c^
C_w_/N_w_	8.0^a^	6.4^b^	5.3^c^	6.6^b^	4.1^d^	4.7^e^	3.2^f^	2.0^g^	1.6^g^	1.2^g,h^	1.1^h^	1.1^h^	1.2^h^	1.1^h^
N–NO^-^_3_ mg kg^-1^	15.0^a^	20.0^b^	15.0^a^	11.0^a^	29.0^c^	209.0^d^	430.0^e^	517.0^f^	603.0^g^	902.0^h^	871.0^i^	992.0^j^	970.0^ k^	982.0^ l^
N–NH^+^_4_ mg kg^-1^	475.0^a^	553.0^b^	242.0^c^	559.0^d^	424.0^e^	244.0^f^	34.0^g^	21.0^h^	8.0^i^	33.0^g^	9.0^i^	5.0^i^	13.0^i,j^	24.0^h^
N_w_ min mg kg^-1^	490.0^nd^	573.0	257.0	570.0	453.0	453.0	464.0	538.0	611.0	935.0	880.0	997.0	983.0	1,006.0
N_w_ org mg kg^-1^	1,272.0^nd^	1,053.0	1,076.0	901.0	429.0	189.0	261.0	301.0	331.0	227.0	163.0	125.0	110.0	112.0

Means followed by the same letter are not significantly different at p < 0.05.

The N_w_ content changed during the composting process (Table 2), significantly decreasing from 1.762 g/kg in the raw material to 0.642–0.725 g/kg after 70–80 composting days and then increasing gradually in the next phases until the end of the process (Table 2). This may be caused by the presence of larger quantities of oxidized forms of nitrogen, i.e. nitrates and nitrites, between the third and fourth months of composting. The duration of municipal waste composting also influenced the C_w_/N_w_ ratio in water extracts (Table 2). In the raw material, the ratio was 8; after 68 days of composting it significantly decreased to 4.7 and in the final phase it reached 1.1. According to many authors^36,38,39^, the C_w_/N_w_ ratio is a better indicator of compost maturity than C/N in the solid phase, showing greater variation depending on the type of initial material. Water-soluble nitrogen occurs in mineral and organic forms. In the first stages of compost maturation (Table 2), N–NO_3_^−^ was present in significantly smaller amounts and N–NH_4_^+^ ions predominated in mineral forms of nitrogen. During the composting process, an irregular but statistically significant decrease in the amount of the N–NH_4_^+^ form was observed, leading to its reduction from 475 mg/kg in the raw material to 24 mg/kg in the mature compost. Said-Pullicino et al.^36^ obtained similar results and this decrease can be explained by transformation into nitrate by bacteria at lower temperatures (below 40 °C). N–NO_3_^−^ forms significantly increased as the compost matured (Table 2), and from about 70 days of composting onwards, a rapid increase in this form of nitrogen was observed. The increase of Nw reflects increase of ammonium and nitrate forms due to microbial mineralization of organic forms of N during composting. According to some authors^40,41^, this describes the phase at which the compost reaches maturity. In the early stages of compost maturation, the N–NH_4_^+^ and N–NO_3_^−^ forms have a weaker influence on the nitrogen soluble in water (Table 2). Total water-soluble nitrogen content (Table 2) was more than doubled—from 490 to 1,006 mg/kg—after composting, due mainly to an increase in N–NO_3_^−^ forms at the end of the process. The organic nitrogen content was significantly reduced during the composting process (Table 2), from 1,272 to 112 mg/kg after composting ended, which indicates the high intensity of the decomposition process that occurs during the composting of organic matter^9^. Change in these parameters during the composting process indicate a favourable course of organic matter transformation^42^. The amount of water-soluble phosphorus (Table 3) significantly decreased systematically during composting, from 113.1 mg/kg in the raw material to 4.5 mg/kg in the matured compost, what is connected with decomposition of organic matter^42^. The water-soluble potassium content did not reduce so rapidly, but a downward trend was also observed. After 9 months of maturation, Ca_w_ content significantly decreased almost tenfold, from 11,840 to 1,670 mg/kg. The less water-soluble magnesium and sodium also significantly decreased during the composting process. The results of this study confirm the research of D’Imporzano and Adani^29^ who observed a rapid decrease of oxygen uptake rate which suggests a limitation of available substrate and lower microbial activity in more mature compost. The strong correlation between nutrient solubility and microbial population activity in composted materials has been also reported by Iglesias and Garcia^43^. Some authors explain change in proportion of water soluble fraction to their total content during composting by change of organic/inorganic fractions of the elements. Wei et al.^44^ noted, that inorganic forms of phosphorus, which prevailed in their experiment with different composts, were solubilized by the acids which were produced during decomposition of organic matter. This was leading to a lower release of phosphorus in solution when compost reached maturity.

**Table 3:** Total content of water extractable macro and microelements in differently matured MSWC.

	Days of composting													
	1	11	22	36	54	68	82	95	112	126	143	159	188	279
**Macroelement mg kg**^**-1**^** d m**														
P_w_	113.1^a^	74.0^b^	57.0^c^	24.0^d^	15.0^e^	19.0^f^	9.0^g^	6.0^g^	6.0^g^	8.0^g^	4.0^g,h^	4.0^g,h^	4.0^g,h^	4.5^g,h^
K_w_	4,660.0^a^	4,020.0^b^	4,630.0^a^	4,690.0^a^	4,390.0^c^	4,490.0^c,d^	4,130.0^b^	3,980.0^b,e^	3,920.0^b,e,f^	3,820.0^f^	3,160.0^g^	3,390.0^h^	3,220.0^g,h^	3,130.0^g^
Ca_w_	11,840.0^a^	10,050.0^b^	3,130.0^c^	2,730.0^c^	1,740.0^d^	1,740.0^d^	1,640.0^d^	1,640.0^d^	1,570.0^d^	1,640.0^d^	1,600.0^d^	1,710.0^d^	1,600.0^d^	1,670.0^d^
Mg_w_	2,880.0^a^	2,630.0^a^	2,210.0^a,b^	1,880.0^b^	1,360.0^c^	1,320.0^c^	1,380.0^b,c,d^	1,330.0^c^	1,350.0^c^	1,330.0^c^	1,250.0^c^	1,200.0^c^	1,200.0^c^	1,400.0^b,c^
Na_w_	3,830.0^a^	3,340.0^b^	4,480.0^c^	3,880.0^a^	3,820.0^a,b^	3,920.0^a^	3,980.0^a^	3,800.0^a,b^	3,670.0^a,b^	3,540.0^a,b^	2,840.0^b^	3,050.0^b^	2,900.0^b^	2,860.0^b,d^
**Microelement mg kg**^**-1**^** d m**														
Cu_w_	2.6^a^	1.9^b^	1.8^b^	1.1^c^	1.5^b,c^	2.3^a,b^	1.9^b^	2.0^b^	1.2^c^	1.6^b^	1.7^b^	1.6^b^	1.6^b^	1.8^b^
Zn_w_	180.8^a^	106.1^b^	46.3^c^	7.0^d^	7.1^d^	9.3^d^	9.3^d^	7.8^d^	8.9^d^	7.9^d^	6.1^d^	5.5^d^	5.3^d^	6.5^d^
Mn_w_	60.0^a^	54.8^b^	6.7^c^	6.0^c^	2.5^d^	2.3^d^	2.0^d^	2.0^d^	2.1^d^	2.2^d^	1.5^d^	1.1^d^	0.9^d,e^	0.7^d,e^
Fe_w_	258.0^a^	239.0^a^	218.0^a^	215.0^a^	166.0^a,b^	182.0^a,b^	144.0^b^	145.0^b^	278.0^a,c^	301.0^a,d^	255.0^a^	273.0^a,c,d^	261.0^a^	317.0^c,d^
Pb_w_	11.6^a^	3.8^b^	3.3^b^	1.9^c^	1.0^d^	1.7^c^	0.6^d,e^	0.6^d,e^	0.8^d,e^	0.6^d,e^	0.6^d,e^	0.4^d,e^	0.6^d,e^	0.5^d,e^
Cr_w_	7.7^a^	2.9^b^	1.8^c^	1.3^d^	0.8^e^	1.2^d^	1.1^d^	1.4^c,d^	1.5^c,d^	1.5^c,d^	1.1^d^	1.6^c,d^	1.6^c,d^	0.5^e^
Ni_w_	7.0^a^	6.2^b^	5.5^c^	3.8^d^	2.7^e^	2.3^e^	1.5^f^	1.8^f^	2.1^e,f^	1.8^f^	1.7^f^	1.1^f,g^	1.1^f,g^	0.1^h^

Means followed by the same letter are not significantly different at p < 0.05.

### Change of WEMIE

The content of most of the water-soluble micronutrients analysed decreased with an increase in composting time (Table 3). The highest amount of water-soluble forms was found in the initial material (fresh) at the beginning of the composting process. Analysis of all the micronutrients studied (Cu, Zn, Pb, Cr, Ni, Mn, Fe) showed that their solubility was usually significantly reduced during the composting process. However, the content of water-soluble Fe was the highest of the components tested and was closest to that in the starting material for the whole study period. The remaining microelements, especially Zn_w_, Mn_w_, Pb_w_, Cr_w_ and Ni_w_, were present in the final composting stage at levels many times and significantly lower than in the raw material. This can be attributed to an increase of humification and pH and/or the formation of stable organo-mineral complexes which are characterized by lower solubility. The solubility of microelements in the soil environment—and in the composts as well—is influenced mainly by pH, CEC and organic matter content^45^. Thus, when decomposition of organic matter and an increase of pH occur during the composting process, the solubility of WEMIE decreases. Moreover, microelements can interact with humic substances, forming complexes and chelates of varying mobility. This ability depends on the structure of humic substances and the amount of functional groups containing oxygen^45,46^. Advanced phases of the composting process are characterized by the formation of stable humic substances with an abundance of aromatic carbon- and oxygen-containing functional groups which can easily interact with microelements^28,47–51^. Hence the amount of WEMIE in more mature compost is much lower than that in the raw material. Similar results were obtained by Leita and De Nobili^30^ who described a rapid decrease of water-extractable fractions of Pb and Zn with an increase in composting time.

### The share of WEMAE and WEMIE in relation to their total content

#### Macroelements

Analysis of the share of soluble forms of macro- and microelements in relation to their total content showed that during composting of municipal waste, soluble macroelements (C, P, K, Ca and Mg) significantly decreased relative to their total forms as maturation progressed. The process was most marked for potassium, magnesium and nickel. The proportion of soluble forms of these components in the final composting phase was below 10, and in the case of phosphorus even below 1. The water solubility of C organic compounds significantly changed during composting and the amount of carbon in the aqueous solution was also affected by the length of the process (Table 4). Dissolved organic matter, represented by C_w_ concentration, contained a high amount of easily biodegradable organic compounds in the first stages of the composting process. After 3 months of composting, dissolved organic matter consists mainly of macromolecules related to stable humic substances^24^, thus during the following stages of the composting process the C_w_ concentration is rather steady without significant differences. A decrease of *I*_*C*_ to below 1 after 112 days of composting may indicate that a period of 3–4 months is sufficient for the production of mature compost. High solubility indices were found for nitrogen, potassium, sodium and magnesium, which may indicate a greater share of the soluble forms of these components in the final phases of compost maturity, in comparison to most other nutrients. The solubility index for iron indicates that the composting process does not affect the degree of solubility of this component.

**Table 4:** Solubility indexes of macro and microelements in differently matured MSWC.

Days of composting								
	1	22	54	82	112	143	159	279
*I*_*C*_	4.93^a^	3.66^b^	2.15^c^	1.43^d^	0.95^d^	0.76^d^	0.87^d^	0.83^d^
*I*_*N*_	27.8^a^	19.3^b^	51.4^c^	64.0^d^	64.9^d^	84.4^e^	88.9^e^	90.0^e,f^
*I*_*P*_	5.95^a^	2.59^b^	0.63^c^	0.38^c^	0.21^c,d^	0.15^c,d^	0.14^c,d^	0.19^c,d^
*I*_*K*_	93.20^a^	85.74^b^	77.02^c^	71.21^c^	67.59^c,d^	50.97^e^	62.78^d^	60.19^d,e^
*I*_*Ca*_	31.74^a^	7.56^b^	3.82^c^	3.64^c^	3.49^c^	3.46^c^	3.42^c^	3.15^c^
*I*_*Mg*_	60.00^a^	40.93^b^	22.67^c^	21.90^c^	22.13^c^	20.16^c^	13.95^d^	16.47^d^
*I*_*Na*_	51.07^a^	71.11^b^	74.90^c^	78.04^c^	78.09^c^	51.64^a^	50.00^a^	44.69^d^
*I*_*Cu*_	2.02^a^	1.33^b^	1.06^b^	1.28^b^	0.79^b^	1.06^b^	0.99^b^	1.05^b^
*I*_*Zn*_	12.28^a^	2.58^b^	0.34^c^	0.45^c^	0.43^c^	0.27^c^	0.24^c^	0.27^c^
*I*_*Mn*_	22.81^a^	2.17^b^	0.70^c^	0.56^c^	0.58^c^	0.41^c^	0.30^c,d^	0.18^c,d^
*I*_*Fe*_	2.95^a^	2.38^b^	1.74^c^	1.46^c^	2.79^a^	2.29^b,c^	2.19^b,c^	2.46^a,b^
*I*_*Pb*_	3.41^a^	0.95^b^	0.28^c^	0.15^c^	0.19^c^	0.13^c^	0.08^c^	0.09^c^
*I*_*Cr*_	17.87^a^	3.92^b^	1.64^c^	2.07^b,c^	2.84^b,c^	2.22^b,c^	3.19^b,c^	0.95^c^
*I*_*Ni*_	30.04^a^	23.50^b^	11.49^c^	5.03^d^	7.78^d^	6.18^d^	3.73^d,e^	0.34^f^

Means followed by the same letter are not significantly different at p < 0.05.

#### Microelements and compost maturity indices

The share of soluble forms of the microelements analysed in relation to their total content also depended on composting time (Table 4). For all micronutrients, a significant reduction in the proportion of soluble forms in relation to their total content was observed with an increase in composting time (Table 4). Except for Fe and Cu, the value of this index in the final composting stage was below 1, indicating the formation of small amounts of water-soluble micronutrients. The highest values of this indicator were found in the raw material. This was especially evident for Ni and Mn, whose indices at the beginning of the experiment exceeded 20, and for Cr (17.9) and Zn (12.3). The results of these studies indicate that the MSWC maturation process also leads to a reduction in the risk of heavy metal presence in the matured compost. The reduction of the proportion of soluble forms of macro- and micronutrients in relation to their total forms can be explained by their incorporation into microbial structures and into complexes with humic substances. The possibility of leaching of the most soluble components from the pile under the influence of water precipitation should also be taken into consideration as a reason for this reduction.

Significant correlation coefficients showing the relationship between composting time and the chemical parameters analysed are presented in Table 5. Some of these dependencies are similar for most of the compost characteristics studied. For example, P_w_, Ca_w_, Mg_w_, Mn_w_, Pb_w_, Ni_w_, N–NH_4_^+^ and C_w_ were significantly negatively correlated with the length of composting time. Some characteristics exhibited a significant positive correlation with composting time: N, N–NO_3_^−^ and pH of aqueous extracts. This indicates the intensive processes of decomposition of organic matter occurring during the composting of municipal waste, which are accompanied by accumulation and transformation of nitrogen and changes in alkaline cations and expressed in the pH increase. It follows that the quality and fertility of the compost depends on the composting process being carried out properly.

**Table 5:** Significant correlation coefficients between water—extractable forms of macro and microelements in MSW compost.

Variable	Days	pH	P_w_	K_w_	Ca_w_	Mg_w_	Na_w_	Zn_w_	Mn_w_	Pb_w_	Ni_w_	C_w_	C_w_/N_w_	N-NO_3_	N-NH_4_
Days	׀														
pH	0.66	׀													
P_w_	− 0.69	ns	׀												
K_w_	− 0.88	ns	0.57	׀											
Ca_w_	− 0.57	− 0.76	0.94	ns	׀										
Mg_w_	− 0.68	− 0.87	0.97	0.56	0.93	׀									
Na_w_	− 0.75	ns	ns	0.91	ns	ns	׀								
Zn_w_	− 0.56	− 0.75	0.97	ns	0.97	0.92	ns	׀							
Mn_w_	− 0.56	− 0.73	0.92	ns	0.99	0.90	ns	0.96	׀						
Pb_w_	− 0.58	− 0.73	0.94	ns	0.89	0.87	ns	0.96	0.86	׀					
Ni_w_	− 0.85	− 0.89	0.94	0.69	0.85	0.95	ns	0.85	0.83	0.83	׀				
C_w_	− 0.75	− 0.85	0.92	0.67	0.87	0.95	ns	0.85	0.84	0.86	0.93	׀			
C_w_/N_w_	− 0.84	− 0.80	0.84	0.84	0.74	0.85	0.59	0.72	0.72	0.76	0.89	0.94	׀		
N-NO_3_	0.90	0.68	-0.67	− 0.91	− 0.53	− 0.69	− 0.76	ns	ns	− 0.55	− 0.80	− 0.78	− 0.92	׀	
N-NH_4_	− 0.75	− 0.70	0.71	0.71	0.67	0.75	ns	0.59	0.66	0.59	0.80	0.87	0.92	-0.86	׀

significant at p < 0.05; ns—not significant.

Numerous authors have worked on the evaluation of compost maturity with reliable indices based on the transformation of organic matter, biological tests, chemical parameters and some others^22,36,40,42,52–58^. Some authors^41,53,59^ point to lack of reference analytical methods as a reason for making comparisons of the final products difficult, especially composts prepared from various organic wastes. In our experiments, compost reached maturity after 82–112 days although the C/N ratio reached a threshold of below 15 after 54 days, thus it was not a good indicator of compost maturity. These findings are in good agreement with those of Wu et al.^23^ who claimed that C/N ratio is not an accurate indicator of compost stabilization and maturation processes. Hence on the bases of our own research, and prior to future research, we propose that the solubility index instead of the content of water-extractable forms of the chosen macro- and microelements should be taken into account for determining the degree of MSWC maturity. The results from the correlation analysis (Table 6) showed that there are at least two indices worth particular attention: *I*_*K*_ and *I*_*Ni*_, which were significantly correlated with composting time. Taking in account other compost parameters, i.e. stabilization of the temperature, stabilization of *I*_*C*_, changes in N–NH_4_^+^ and N–NO_3_^−^, it can be concluded that the compost reached maturity after 82–112 days. The solubility index for potassium, indicating the maturity of the composted material, reached a value below 75; for nickel, it was below 10. Future investigations in this direction, with different kinds of composted materials, may bring interesting solutions.

**Table 6:** Significant* correlation coefficients between composting time and solubility index of some macro and microelements.

Variable	Composting days	*I*_*P*_	*I*_*K*_	*I*_*Ca*_	*I*_*Mg*_	*I*_*Cu*_	*I*_*Zn*_	*I*_*Mn*_	*I*_*Pb*_	*I*_*Cr*_
*I*_*K*_	− 0.81	0.83								
*I*_*Ca*_		0.96	0.72							
*I*_*Mg*_	− 0.74	0.98	0.86	0.92						
*I*_*Cu*_		0.94	0.75	0.92	0.90					
*I*_*Zn*_		0.97	0.74	0.99	0.93	0.93				
*I*_*Mn*_		0.95		0.99	0.89	0.91	0.99			
*I*_*Pb*_		0.99	0.77	0.99	0.95	0.93	0.99	0.99		
*I*_*Cr*_		0.94		0.99	0.89	0.89	0.99	0.99	0.98	
*I*_*Ni*_	− 0.83	0.93	0.89	0.82	0.97	0.81	0.84	0.79	0.88	0.80

*significance level < 0.05

## Summary

Mobility and availability of macro and micro nutrients to plants, their sorption and desorption are affected by numerous factors like plant species, soil pH, content of organic matter, calcium carbonate and chemical properties of given element. Stabilized insoluble organic matter in a form of matured compost, when applied to the soil induces binding of metals and metalloids and therefore their immobilization. Low molecular organic substances present in unmatured product like organic acids, polyphenols cause either complexing or chelating of metals or metaloids, and the resulting bonds are characterized by movement throughout the soil^60,61^. Characteristics of organic matter will be the main object of the manuscript that is preparing yet for publication thus in this experiment authors focused on analysis of the water-soluble forms of macro- and microelements. Based on the results obtained, it can be concluded that the tested compost reached maturity after about 70 days of the composting process.

The share of soluble forms of the microelements analysed in relation to their total content depended on composting time. During the composting of municipal waste, the content of soluble macronutrients (C, P, K, Ca and Mg) relative to their total forms decreased as maturation progressed. For all micronutrients, a significant reduction in the proportion of soluble forms in relation to their total content was observed with an increase in composting time. The amount of WEMIE in more mature compost was much lower than that in the raw material. The *I*_*C*_ index may indicate that a period of 3–4 months is sufficient for the production of mature compost. In mature compost, high solubility indices were found for nitrogen, potassium, sodium and magnesium, which may indicate that the final product is a good source of these nutrients. After applying mature compost to the soil, these components will be easily available for plants. The solubility index for iron indicates that the composting process does not affect the degree of solubility of this component. The solubility index for potassium, indicating the maturity of the composted municipal waste, reached a value below 75; for nickel, it was below 10.
